# Biomarkers of Angiogenesis and Neuroplasticity as Promising Clinical Tools for Stroke Recovery Evaluation

**DOI:** 10.3390/ijms22083949

**Published:** 2021-04-11

**Authors:** Lidia Wlodarczyk, Rafal Szelenberger, Natalia Cichon, Joanna Saluk-Bijak, Michal Bijak, Elzbieta Miller

**Affiliations:** 1 Department of Occupational Diseases and Environmental Health, Nofer Institute of Occupational Medicine, 91-348 Lodz, Poland; lidia.monika.wlodarczyk@gmail.com; 2 Department of General Biochemistry, Faculty of Biology and Environmental Protection, University of Lodz, Pomorska 141/143, 90-236 Lodz, Poland; rafal.szelenberger@edu.uni.lodz.pl (R.S.); joanna.saluk@biol.uni.lodz.pl (J.S.-B.); 3 Biohazard Prevention Centre, Faculty of Biology and Environmental Protection, University of Lodz, Pomorska 141/143, 90-236 Lodz, Poland; michal.bijak@biol.uni.lodz.pl; 4 Department of Neurological Rehabilitation, Medical University of Lodz, Milionowa 14, 93-113 Lodz, Poland; elzbieta.dorota.miller@umed.lodz.pl

**Keywords:** stroke, recovery, biomarkers, prognosis, personalized medicine, rehabilitation

## Abstract

Several key issues impact the clinical practice of stroke rehabilitation including a patient’s medical history, stroke experience, the potential for recovery, and the selection of the most effective type of therapy. Until clinicians have answers to these concerns, the treatment and rehabilitation are rather intuitive, with standard procedures carried out based on subjective estimations using clinical scales. Therefore, there is a need to find biomarkers that could predict brain recovery potential in stroke patients. This review aims to present the current state-of-the-art stroke recovery biomarkers that could be used in clinical practice. The revision of biochemical biomarkers has been developed based on stroke recovery processes: angiogenesis and neuroplasticity. This paper provides an overview of the biomarkers that are considered to be ready-to-use in clinical practice and others, considered as future tools. Furthermore, this review shows the utility of biomarkers in the development of the concept of personalized medicine. Enhancing brain neuroplasticity and rehabilitation facilitation are crucial concerns not only after stroke, but in all central nervous system diseases.

## 1. Introduction

According to the updated definition, stroke is an acute episode of neurological dysfunction caused by the cerebral, spinal cord, retinal infarction, or hemorrhage (including subarachnoid hemorrhage). The medical state may persist longer than 24 h or of any duration, if focal infarction or hemorrhage relevant to the symptoms has been shown by imaging (like CT or MRI scans) or autopsy [1]. As a medical condition, stroke is a complex, heterogeneous disease in terms of semiology, etiology, and possible treatment strategies. The clinical symptoms are directly related to the topography of brain tissue damage and commonly include paresis (most frequently diagnosed), numbness, speech disorder (i.e., aphasia, dysarthria), vision impairment, and disturbed coordination [2]. There are two main causes of stroke associated with its etiology: ischemia and hemorrhage. Ischemic stroke may develop from various origins, including embolism (arterial or cardiac in origin), decreased perfusion (arterial occlusion or stenosis), and thrombosis (as a primary or secondary abnormality of hemostasis). Focal ischemia leads to irreversible injury in a core region and partially reversible damage in the surrounding penumbra zone. In contrast, hemorrhagic stroke is associated with the leakage or rupture of the artery and remains in a minority—approximately only 15% of strokes are diagnosed as hemorrhages. Despite the separation of the stroke into two main causes, the proportions of pathological and etiological subtypes differ depending on age, race, ethnic origin, and nationality. Although the mortality rate from a stroke has decreased over the recent years, it remains the main cause of disability within professionally active people over 40 years of age [3]. There are quite effective early treatment strategies for ischemic strokes, like intravenous tissue plasminogen activator (tPA) or endovascular thrombectomy (EVT) [4]. Patients are eligible for medical thrombolysis (intravenous administration of tissue plasminogen activator) within 4.5 h of known symptom onset (in various situations, the thrombolysis time window may extend beyond 4.5 h). The time window for EVT treatment is available for all patients who arrived at the hospital within 6 h and, in particular cases, within 6–24 h (including stroke upon awakening with unknown onset time). All acute stroke patients, who are not receiving tPA or EVT therapy, should be administrated with acetylsalicylic acid (ASA) [5]. Furthermore, antiplatelet drugs should be continued as secondary ischemic stroke prevention. Treatment of hemorrhage stroke focuses on reducing intracranial and blood pressure, preventing seizures and vessel spasm. Moreover, surgical hematoma drainage should be considered if necessary [4,6]. In every case, the initial treatment is aimed at improving basic vital functions by the connection of a drip and oxygen. During acute inpatient care, stroke patients should also undergo investigations to determine stroke mechanisms, guide stroke prevention, and future management decisions. To provide medical care, an interdisciplinary medical team on the stroke unit should be set up, thus providing a suitable evaluation of the patient’s health condition and enabling the selection of an appropriate type of post-stroke rehabilitation, which should be implemented immediately after the patient’s condition stabilization. Rehabilitation therapy should begin as soon as the patient can participate in physical exercises. The rehabilitation process should be directed at affected functioning domains including motor impairments, speech disorders, cognitive dysfunction, vision disturbances, etc. Post-stroke rehabilitation uses selected therapies for motor function improvement like aerobic training (AE), repetitive task training (RTT), constraint-induced movement therapy (CIMT), mirror therapy, muscle strengthening, electromechanical, robot-assisted training, noninvasive brain stimulation (NIBS), and neuromuscular electrical stimulation (NMES) [7]. To maximize the post-stroke care effectiveness, coordinated effort from the medical team is required including physical and occupational therapists, speech-language pathologists, psychologists, nutritionists, recreation therapists, and others [8].

The main goal of this review is to introduce a new approach for stroke recovery biomarkers, which are based on angiogenesis and neuroplasticity processes. The proteins present in the manuscript could be used to determine the innovative algorithm of the procedure to evaluate an outcome of post-stroke patients and applied therapy.

## 2. The Definition of a Biomarker

Acute damage of neural matter results in the inflammatory reaction, loss of function and neurodegeneration, but functional recovery and structural rebuilding processes are stimulated concomitantly. Therefore, it is essential to develop and implement the biomarkers responsible for effective and successful brain recovery. The classic definition for biomarker was given by Naylor, and assumed that it should be objectively measured and evaluated as a marker of both physiological and pathological processes, and also as an indicator of response to a therapeutic intervention [9]. Thus, biomarkers can play a role in identifying risk factors of the disease, screening, diagnosing, and also making a prognosis. To assess a potentially useful biomarker, several criteria must be fulfilled. To correctly identify a high proportion of true positive or true negative rate, biomarkers must be sensitive and specific. The collection process must be safe and non-invasive, measurements must be repeatable, and all methodology should be cost-effective, quick, and not interfering with applied therapies [10].

In turn, a stroke recovery biomarker may be defined as an indicator of outcome or expected improvement, as well as treatment response after ischemic or hemorrhage brain tissue damage. Moreover, it is important to distinguish prognostic biomarkers that are based on, inter alia, demographic data, clinical assessments, and natural history of the disease, from predictive biomarkers, that is a determinant of response or non-response to a specific therapy [11]. The division of markers into prognostic and predictive ones was done analogously according to the definition proposed by Clark [11]. Therefore, the prognostic factor is a measure of recovery and outcomes resulting from standard management, and the predictive factor implicates a diverse benefit from particular treatment [11]. Furthermore, stroke recovery biomarkers, useful for personalized rehabilitation planning, should predict outcomes for specific functional domains, because using general outcome (good or poor) measures like modified Rankin Scale (mRS) may be insufficient to predict results meaningful for individuals [12,13].

## 3. Angiogenic Biomarker of Stroke Recovery

### 3.1. Clinical (Physiological) Aspects of Angiogenesis

Angiogenesis is the physiological process that denotes the formation of new blood vessels from currently existing vasculature [14,15]. The major role of angiogenesis is to create a network for remodeling of arteries (arteriogenesis) and veins (vasculogenesis) during embryogenesis, and/or wound healing. For this purpose, the angiogenic growth factors and inhibitors are mixed and coordinated to induce and sustain the migration and proliferation of endothelial cells [16]. The mechanism of angiogenesis is comprised of three main stages. Firstly, among endothelial cells, vascular endothelial growth factor (VEGF-A) with Notch family receptors and their ligands, select and empower endothelial cells to proliferate and protrude filopodia, and become “tip cells”. With the increased mobility, “tip cells” are able to induce the invasion and migration towards the angiogenic stimulus. Next, the migration and proliferation of endothelial cells is enhanced and mediated by the growth factor-receptor interaction (VEGF-A—VEGFR-2). The third stage assumes maturation of newly sprouted vessels, the inhibition of angiogenesis by reduction of proliferation and migration of new capillaries, and supporting the connection of new vessels with pre-existing ones [15]. In pathological states, angiogenesis is stimulated by hypoxia, which is one of the major stroke-related medical conditions associated with reduced oxygen levels in the blood. Studies have shown that angiogenesis genes are highly expressed within minutes after ischemic stroke in rodents, and the concentration of released angiogenic proteins is elevated and lasts for days or weeks in the ischemic area [17]. Similarly, an increased level of angiogenic proteins has been detected in humans, indicating positive and negative feedback on the patient’s recovery. Furthermore, an autopsy study performed by Krupinski et al. showed that in the penumbral region, increased density of microvessels can be observed [18]. This finding was also correlated with their longer survival [19]. Interestingly, intense neovascularization was observed in younger patients, indicating the reduction of angiogenesis in older patients. Another major finding showed that not pharmacologically stimulated angiogenesis was progressing for only 3–4 days after stroke [20], suggesting the possibility of its monitoring to evaluate the widening of a therapeutical window in response to therapy. The beneficial role of angiogenesis is well known. However, scientists assume that neovascularization may have more neurorestorative than neuroprotective nature [21]. In a study conducted by Shen et al., combined cell culture of neural stem cells and endothelial cells indicated a critical role of secreted soluble factors from endothelial cells to maintain self-renewal of central nervous system stem cells, and their neurogenic potential. Furthermore, coculture provided symmetric and proliferative division of neural stem cells to produce undifferentiated stem cell sheets, generate neurons, oligodendrocytes, and astrocytes [22]. These results show that examination of vascularization in post-stroke patients may be a good biomarker for specific patients to monitor the effect of pharmacotherapy and rehabilitation. Here, we would like to introduce biochemical biomarkers of angiogenesis and imaging techniques that could assess a patient’s recovery outcome.

### 3.2. Biochemical, Angiogenic Biomarkers of Stroke Recovery

VEGF-A, usually named VEGF, is a protein that is well known to regulate and induce angiogenesis in various pathological conditions, including embryogenesis [23]. In the case of ischemic stroke, the *VEGF-A* gene expression is upregulated due to hypoxia, and synthesized protein usually binds to VEGFR-2, which is a major mediator of angiogenesis, mitogenesis, and enhanced permeability induced by VEGF-A. The second receptor, may induce the stimulation of matrix metalloproteinases (MMPs) and the release of endothelial growth factors [24]. Neurons, glial cells, and migrated macrophages, which also participate in angiogenesis, are the main source of released VEGF during the cerebral ischemia. The deteriorating state of the neovasculature microenvironment causes the production of pro-inflammatory cytokines, which has the ability to modulate *VEGF* expression, thus enhancing the concentration of VEGF and stimulating the angiogenesis [25]. Studies on animal models show that VEGF not only stimulates angiogenesis but is also associated with elevated transdifferentiation of reactive astrocytes into neuroprogenitors and new neurons [26]. VEGF-A expression in the acute phase of ischemic stroke was shown to be significantly elevated [27,28] and persist for even 90 days after stroke onset [27]. Furthermore, Matsuo et al.’s study showed that VEGF level during the examination was not associated with risk factors like hypertension, dyslipidemia, diabetes, atrial fibrillation, smoking, and alcohol [27]. The same study examined VEGF values by multivariate logistic regression analysis to assess its role as a predictor of functional outcome. Results showed that higher values of VEGF on the stroke-occurred-day may predict poor outcomes for patients with a cardioembolic infarction origin stroke [27]. Serum VEGF level was also associated with post-stroke cognitive impairment. Pordjohardjono et al. showed that a higher VEGF level was linked with an almost 5-fold higher incidence of cognitive impairment. Interestingly, cognitive impairment was positively associated with larger infarct volume [29]. On the other hand, the impact of rehabilitation on VEGF concentration was determined in a study conducted by Ma et al. Results showed that running significantly elevated the level of VEGF and MMP2, and augmented the absolute regional cerebral blood flow in the ischemic area 16 days after stroke in animal models, thus having beneficial effects on long-term recovery [30]. The discrepancy between described studies is not fully understood, especially while many studies indicate the positive, neuroprotective role of VEGF. The possible explanation could be associated with the early VEGF concentration and the severity of stroke. The correlation between higher level of VEGF and the augmented infarct volume [29] could confirm this hypothesis, indicating that the release of higher amount of VEGF is essential for reducing the consequences of an increased-damage zone in the brain tissue, thus not leading to deleterious effects. However, to confirm that hypothesis, further investigations are required.

Taking into consideration all the above-mentioned evidence, it could be suggested that higher VEGF levels may indicate the extent and complexity of damage caused by cerebral ischemia. The confirmation of this hypothesis could be very helpful for clinicians and neurologists to assess the severity in the acute phase of the disease. Furthermore, long-term monitoring of VEGF level could be very helpful to define the patient’s chance for a good outcome and to verify the successfulness of used pharmacotherapy and rehabilitation.

The effect of angiogenesis may be enhanced by other angiogenic factors, including angiopoietins (Angs), which represent an important superfamily of growth factors [31]. The angiopoietins bind with one of two tyrosine kinase receptors, Tie1 or Tie2, whose expression is almost solely found in hematopoietic stem cells and endothelial cells [32]. Ang-1 is a direct agonist of the Tie2 receptor and is responsible for maintaining vasculature, while Ang2 serves as an antagonist, whose main function is linked with angiogenesis [31]. Both multimeric ligands bind with Tie2 with similar affinity. However, Ang2 leads to a much weaker activation of Tie2. Ang2 is expressed exclusively in endothelium and unlike Ang1, its production is controlled by the level of different growth factors or pathological conditions, including hypoxia or VEGF-A. The expression of Ang2 in quiescent vasculature is undetectable, although angiogenic activation of endothelium causes high upregulation and rapid synthesis of Ang2, thus promoting angiogenesis. The Ang-Tie signaling system is essential for physiological functioning. Its understanding in vascular biology is, however, poorly understood. Experimental studies showed that Ang2 can also act as an agonist for Tie2, by phosphorylating tyrosine residues, although certain biological processes must be carried out. The phosphorylation of cytoplasmic tyrosine residues of Tie2 by Ang1 causes the stimulation of several intracellular signaling pathways, including nitric oxide synthesis stimulated by protein kinase B, mitogen-activated protein kinase pathways, phosphoinositide 3-kinase and protein kinase B pathway (PI3K/AKT). The activation of Tie2/PI3K/AKT pathway leads to the inhibition of transcription factor FOXO1 which results in the low expression of Ang2. In contrast, low AKT activity results in the activation of FOXO1, which causes the elevation of Ang2 expression, thus enabling the phosphorylation of Tie2 by Ang2 [33]. Experimental studies evaluating the role of Ang2 in inflammations showed that its blockage resulted in inhibition of vascular remodeling and reduced changes in endothelial phenotype, including the expression of E- and P-selectin [34]. In a mouse model of occlusion of the middle cerebral artery, the mRNA expression of *Ang2* in the ischemic cortex was four times higher in comparison to the control, contralateral healthy cortex. In addition, intracerebroventricular injection of Ang2 led to a significant decrease in vascular leakage, indicating that Ang2 may reverse blood–brain barrier (BBB) permeability induced by VEGF [31]. Furthermore, in a study performed by Moisan et al. that evaluated the microvascular plasticity after experimental stroke, *Ang2* was overexpressed in the acute stage of stroke, suggesting that its augmented level was linked with a detachment of pericytes, vasodilatation, and destabilization of vessels. The second peak of *Ang2* was observed in the subacute stage, 7 days after onset of symptoms, and its role was associated with the promotion of endothelial cell survival and stabilization of vasculature [35]. Another animal model study showed that physical exercise caused a significant augmentation in the expression of the Tie2 receptor. Early exercises reduced infarct volume and improved functional outcomes, thus suggesting an important role of angiopoietin receptor, which is consistent with the theory of Ang2-Tie2 role in improving cerebral blood flow in ischemia cortex, and enhancing microvasculature [36]. Studies based on the potential use of Ang2 as a biomarker of stroke recovery were performed in the vast majority on animal models. Furthermore, Ang2 or Tie2 levels in plasma were not evaluated in post-stroke patients. However, in a study conducted on patients with coronary heart disease, Ang2 level was upregulated, thus suggesting its dysregulation in cardiovascular disorders [37]. The above-mentioned results showed that Ang2 and/or Ang2-Tie2 signaling may be very promising for monitoring stroke severity and functional outcome in humans, especially if the protein concentration and gene expression levels could be determined in the diagnostic panel with simultaneous analysis of imaging techniques. However, this hypothesis should be confirmed in further investigations.

## 4. Neuroplastic/Neurogenic Markers of Stroke Recovery

### 4.1. Clinical (Physiological) Aspects of Neuroplasticity and Neurogenesis

Neuroplasticity is defined as a process involving adaptive structural and functional changes in the brain in response to intrinsic or extrinsic stimuli. The neuroplastic process is related to synaptogenesis, neurogenesis, and neuroprotection. In a stroke, neuroplasticity begins immediately after an ischemic event under the critical conditions of inflammation, edema, metabolic disturbances, apoptosis, and nerve fiber degeneration [38]. Neuroplasticity is a very complex process, which remains unclear and relies on the consolidation of existing synaptic pathways to create new connections. The remaining, but diminished connections between brain centers are activated. Consequently, the defective function can be restored when other cortical or subcortical structures take over the function of the damaged area; as a result, it leads to spontaneous limited recovery in post-stroke patients. Stroke Recovery and Rehabilitation Roundtable clearly defined the time frame of the post-stroke recovery. Additionally, the following phases were distinguished: hyper-acute (0–24 h), early subacute (7 days–3 months), late subacute (3–6 months), and chronic (>6 months), indicating that the critical period for neuroplasticity is in the acute and early subacute phases [39]. In the acute phase of a stroke, secondary neuronal networks are used to maintain function, whereas in the subacute phase, new synaptic connections are created, and in the chronic phase, remodeling by axonal sprouting and further reorganization occurs [40]. All those processes can be positively influenced by several factors, such as the environment, repetition of tasks, motivation, neuromodulators, medications, and more.

Neurogenesis is a process in which new nerve cells increase during the differentiation of neural stem/progenitor cells (NSPCs). It is more likely during global ischemia when the selective death of vulnerable neuronal populations, like the pyramidal neurons of hippocampal CA1, occurs. It has been reported that ischemic insult induces the creation of new neurons in the adult rodent brain from NSPCs located in two regions: near the lateral ventricle (subventricular zone) and in the dentate gyrus (subgranular zone) [41]. Ischemia-induced neurogenesis is also triggered in the nonneurogenic area in the undamaged brain, for example the striatum [41,42]. So far, there is limited knowledge about the prevalence of neurogenesis in the brain of post-stroke patients, mainly due to the need for post mortem analysis. Nevertheless, Marti-Fabregas et al. showed that in post-stroke patients, an increased number of NSPCs in the ipsilateral subventricular zone, as well as augmented proliferative activity, were present [43]. In turn, Macas et al. confirmed the regenerative capacity of the adult human brain in response to ischemia that persisted in the elderly [44].

### 4.2. Biochemical Neuroplastic Biomarkers of Stroke Recovery

Brain-derived neurotrophic factor (BDNF) is one of the most detailed studied neurotrophic factors belonging to the family of neurotrophins, which also includes nerve growth factor (NGF), BDNF, neurotrophin-3 (NT-3), and neurotrophin-4 (NT-4) [45]. BDNF is involved in neuroprotection, neurogenesis, and the adaptation of synaptic activity. BDNF, by binding to the receptor tyrosine kinase B (TrkB) and activating several signaling pathways: Akt/PKB, phosphoinositide 3 kinase (PI3K), as well as Insulin Receptor Substrate 1 or 2 (IRS1/2), promotes protein synthesis, cell survival, dendritic maturation, axonal growth, synaptic plasticity, stress resistance, and therapeutic neovascularization [46,47]. BDNF also plays a key role in mediating the maintenance of long-term potentiation (LTP) of glutamatergic synapses, mainly with hippocampal synapses playing a role in episodic memory, but also other brain areas, including the primary motor cortex [48,49]. BDNF may be a useful post-stroke recovery marker due to its broad neurotrophic activity, as well as changes in its plasma concentration during recovery. The correlation between low serum BDNF levels in the first day after stroke and the poorer long-term functional prognosis is well documented [50,51,52]. Navarro-Martínez et al. observed that the BDNF plasma concentration positively correlated with the functional status (measured by the Barthel scale) as well as with the cognitive functions (assessed by the Mini–Mental State Examination (MMSE scale)) in post-stroke patients [53]. Lasek-Bal showed that patients with BDNF levels in the acute phase below the mean (9.96 ng/mL ± 5.21 ng/mL) functional status (according to the National Institutes of Health Stroke Scale (NIHSS) and mRS) 90 days after the ischemic event had a significantly worse outcome than the group of patients with higher BDNF levels [50]. On the other hand, Stanne et al. found that a decreased BDNF level after hospital admission was associated with a poor functional outcome in both two years (score 3–6 on the mRS scale) and seven years (3–5 mRS) after stroke [52]. Moreover, it has been shown that the level of BDNF increases significantly after physical rehabilitation, regardless of the type of therapy [54,55]. Koroleva et al. demonstrated that active rehabilitation favored the increase of BDNF and functional improvement of patients; while in patients without rehabilitation, the BDNF concentration tended to decrease [55]. Moreover, it has also been shown that an increase in BDNF levels was conducive to the improvement of post-stroke depression, including estrogen therapy [56] and physical rehabilitation [57]. The utility of post-stroke therapy monitoring was demonstrated by Chan et al. and the results showed the correlation between a greater increase in BDNF and greater functional post-stroke improvement in patients 6 months after the ischemic event [58]. The BDNF cut-off point in post-stroke disability or recovery has not been clearly established. Lou et al. found that a cut-off value of 20.7 ng/mL (specificity of 62.3%; sensitivity of 51.7%) could be an optimal value for short-term post-stroke recovery prediction [59], whereas Stanne et al. suggested a cut-off value of 21.8 ng/mL as a good predictor for a long-term recovery [52]. Importantly, the level of BDNF does not differ between sex, age and the initial severity of stroke. A significant increase in the plasma level of BDNF associated with a better therapeutic prognosis suggests that monitoring its concentration during therapy may allow assessment of the functional progress. Nevertheless, BDNF as a biomarker has some limitations, because the Val66Met polymorphism of the *BDNF* gene is associated with worse post-stroke recovery, regardless of the total plasma concentration [60].

Another protein of prognostic interest in post-stroke recovery is fibronectin type III domain-containing protein 5 (FNDC5), usually called irisin. Irisin constitutes one of the most important myokines secreted mostly by skeletal muscles and adipose tissue [61], whose concentration increases due to physical activity [62]. The increased concentration of irisin is correlated with increased expression of BDNF. Furthermore, FNDC5 was found to reduce neuronal damage caused by oxidative stress by reducing the secretion of pro-inflammatory cytokines, and augment the expression of superoxide dismutase, glutathione peroxidase, and catalase 9 to reduce the production of harmful reactive oxygen species during inflammation [61]. Irisin was also associated with depression in post-stroke patients. In a study conducted by Tu et al., serum level of irisin was lower in patients with depression in comparison to patients without depression (*p* < 0.001) [63]. Kim et al. performed a study in which an increased level of FNDC5 was positively correlated with physical exercise in both mice and human subjects [64]. Moreover, the mechanism of action was also suggested in the prevention of cardiovascular diseases. In a study conducted by Song et al., irisin induced the proliferation of Human Umbilical Vein Endothelial Cell (HUVEC) through the extracellular signal-related kinase (ERK) pathway. Furthermore, it showed protective properties from high glucose-induced apoptosis by regulating caspase expression [65]. In a stroke, irisin may be a useful early predictor. Wu et al. attempted to define its effectiveness in early functional outcomes in post-stroke patients. Results showed significant negative correlations between the concentration of irisin and NIHSS. In addition, patients who on admission had irisin levels in the first quartile (Q1; lowest irisin levels) had a 94% increased risk of poor outcomes three months after the ischemic episode (odds ratio (OR) for Q1, 1.94 (95% confidence interval (CI), 1.19–3.42); *p* = 0.018) and mortality augmented by 66% (OR for Q1, 1.66 (95% CI, 1.11–3.07); *p* = 0.009) [66]. Similar prognostic data were presented by Tu et al., showing an increase in the risk of poor outcomes by 58% (OR for Q1 1.58 (95% CI, 1.12–2.43)) and mortality by 185% (OR for Q1, 2.85 (95% CI, 1.79–4.02)) of stroke [67]. As mentioned above, FNDC5 shows a significant number of pro-health properties, especially important for post-stroke patients, thus indicating that dysregulation in its concentration may constitute an ideal indicator for monitoring the improvement of health conditions in post-stroke patients.

Matrix metalloproteinase 9 (MMP9) belongs to the family of zinc-dependent endopeptidases associated with pathological and physiological tissue remodeling, including neovascularization. An upregulated level of MMP9 was noticed during inflammation, thus contributing to diseases’ progression and stimulation of immune response, and in contrast, in wound healing. MMP9 is mostly released from neutrophils, macrophages, and fibroblasts in various biological processes, including stimulation via reactive oxygen species, and activated NF-κB (nuclear factor kappa-light-chain-enhancer of activated B cells) pathway [68]. Studies showed that post-stroke patients presented an upregulated level of MMP9 in comparison to healthy donors, which correlated with a poorer outcome [69]. Abdelnaseer et al. noted that elevated levels of MMP9 (measurements were carried out within 24 h of stroke onset) were associated with more severe stroke (according to NIHSS scale) regardless of other variables such as gender, age, and comorbidities (hypertension, diabetes, hyperlipidemia, etc.) [70]. In a study conducted by Rodriguez-Yanez et al., MMP9 was linked with the worsened neurological outcome at 3 months post-stroke patients [71]. A large prospective study by Zhong et al. showed that high levels of serum MMP9 in the acute phase of stroke were correlated with an increased risk of death or severe disability. The optimal cut-off point for MMP9 was established at 812.2 ng/mL. There was a strong linear relationship between the level of MMP9 and functional status in the assessed mRS scale 3 months after stroke (*p* < 0.001) [72]. Hemorrhagic transformation is a serious complication of acute ischemic stroke, resulting in an unfavorable prognosis and delay in anti-mortem treatment. A meta-analysis of 1,492 patients with acute stroke showed a high predictive value for hemorrhagic transformation of MMP9, with 79% specificity (95% CI: 75–91%) and 85% sensitivity (95% CI: 75%, 91%) [73]. The predictive value of MMP9 was confirmed in a recent cohort study presented by Mechtouff et al. Researchers showed that the highest increase in MMP9 concentration was recorded 6 h after the stroke onset, and augmented level of MMP9 was correlated with infarct growth (OR 3.43 (1.23–9.55); *p* = 0.02) as well as hemorrhagic transformation (OR 2.48 (1.16–5.27); *p* = 0.02) [74], thus suggesting that MMP9 may be a useful marker with an ability to exclude patients at high risk of bleeding complications. The summary of selected biomarkers is presented in Table 1.

It is a very important issue to be aware of advantages as well as limitations of biomarkers discussed in the manuscript. A critical appraisal of potential stroke recovery biomarkers might allow for more appropriate and adjusted studies in the future. The number of studies supporting biochemical biomarkers in stroke recovery is severely limited. The low number of experimental studies performed on biological material obtained from humans does not provide a sufficient strength of analysis. Usually, experiments are performed on small sample size, which has a big impact for reproducibility and repeatability of data. Moreover, small population size may generate mutually exclusive results. The vast majority of available researches were performed on animal models. A constantly growing database of publications focused on the searching of stroke biomarkers is unfortunately still relatively scarce, which limits the possibility of combining more than one biomarker in potential diagnostic panels. It is especially important, because one single biomarker might not be specific enough for diagnosis. Furthermore, most studies are focused on an acute phase of stroke, thus strongly reducing the current state of knowledge about subacute and chronic phase of stroke, which is also very important in post-stroke rehabilitation.

In conclusion, there is a need to conduct longitudinal studies with big sample sizes, analyzing biochemical panels as stroke recovery biomarkers in the future.

## 5. Future Research and Directions in Personalized Medicine and Rehabilitation in Stroke Recovery—Discussion

Future studies would be necessary and valuable, as there is no single perfect biomarker of stroke recovery. The value of post-stroke recovery prediction might be built based on multimodal assessment, especially in the context of personalized medicine. Due to the heterogeneity of the central nervous system, the use of single plasma markers has limited clinical application. Therefore, to increase their diagnostic value, the use of a set of panel tests was proposed. Rapid and common diagnostic tests would help select patients for a time-limited treatment strategy. Several panels are currently proposed, one of which is the Triage Stroke Panel. A 10-year clinical study completed in 2015 (NCT00206908), the preliminary results of which are very opportune, showed the clinical usefulness of a rapid immunoassay evaluation of the concentration of S100B, MMP9, fibrin degradation products containing D-Dimer, and B-type natriuretic peptide (BNP) in plasma or whole blood. It was shown that this panel presented high sensitivity in distinguishing ischemic stroke from mimic (c = 0.69; *p* < 0.001), and these results also correlated with the severity of stroke (according to the NIHSS scale) [75]. A biochemical and genetic panel, developed for the rapid determination of the gene expression and protein concentration involved in angiogenesis and neuroplasticity, performed on plasma or whole blood samples, could be carried out in the future for every patient admitted to the hospital. The current state of knowledge allows for laboratory tests to be performed, enabling the creation of a complete picture of molecular disorders in a given disease entity, in various dimensions, providing a better understanding of the pathological features affecting the disease microenvironment, and facilitating development of new insights that could help in better understanding the disease progression. The regulation of biological processes responsible for a cellular response, such as neuronal development, apoptosis, differentiation, proliferation, neuroplasticity, neurodegeneration, and other physiological and pathological conditions, may be implicated within the functionality of microRNA (miRNA) [76]. Due to the important role in the regulation of biological processes, unique changes in miRNA profiles are considered to be potentially useful in reflecting the pathological state of a disease, thus serving as a biomarker. Several miRNAs were found to be associated with post-stroke angiogenesis, neurogenesis, neuroprotection, and suppression of oxidative stress and inflammation [77]. However, further investigation is necessary to confirm results from the previously conducted study and elaborate on a potential diagnostic panel of circulating miRNAs, which show high stability in body fluids and remarkable resistance to harmful conditions, established through laboratory examinations, including a wide range of pH, prolonged storage, the activity of endogenous RNases and repeated freeze-thaw cycles [78]. Furthermore, the combination of biochemical and genetic tests with imagining techniques could help in determining the actual health condition of patients and assess the progress of the applied pharmacotherapy and rehabilitation.

In future studies, post-stroke repercussions that can substantially affect post-stroke recovery, like depression or seizures, should also be remarked on and considered. Currently, depression is diagnosed by clinical scales, and epilepsy is recognized based on medical history, neuroimaging, and neurophysiological investigations. Post-stroke depression is considered to be the most common neuropsychiatric complication of stroke, which is defined as a depressive episode that develops in a causal and temporal relationship with a stroke. Most patients develop symptoms of depression within 3–6 months after a stroke, because of which, it is sometimes not recognized nor treated [79]. Che et al. showed a strong correlation between the level of anticardiolipin antibodies, anti-phosphatidylserine antibodies, MMP9, growth differentiation factor-15 and post-stroke depression (3 months after the ischemic episode), which suggests that the analysis of a panel of several biomarkers from different pathophysiological pathways can be useful in predicting depression [80]. Seizures due to vascular damage to the brain can occur during or immediately after a stroke (so-called early seizures). They can also appear as a late complication of a stroke, after several weeks or months [81]. Abraira et al. proposed a panel of 3 markers (out of 14 tested proteins) for the prediction of post-stroke epilepsy. They showed that a decreased level of heat shock 70 kDa protein-8 (Hsc70) (<2.496 (*p* = 0.006, hazard ratio (HR) 3.795, 95% CI 1.476–9.760), increased level of endostatin (>1.203 (*p* = 0.046, HR 4.300, 95% CI 1.028–17.996) and a low level of S100B (<1.364 (= 0.001, HR 2.955, 95% CI 1.534–5.491) were associated with the incidence of epilepsy. Importantly, the combination of biomarkers with clinical variables compared to them being used alone was associated with a greater predictive ability (74.3%, 95% CI 65.2%–83.3% vs. 68.9%, 95% CI 60.3%–77.6%) [82].

**Figure 1:**
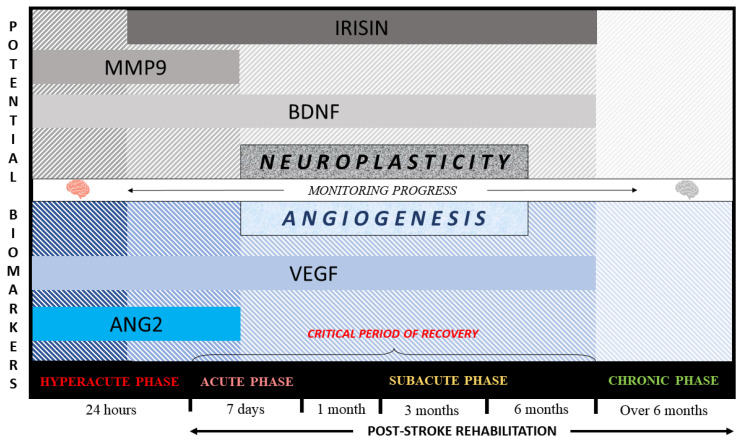
Concept of potential biomarkers for post-stroke rehabilitation. VEGF—vascular endothelial growth factor; Ang2—angiopoietin 2; BDNF—brain-derived neurotrophic factor; MMP9—matrix metalloproteinase 9.

Personalized rehabilitation in clinical practice is a promising tool to improve the ability to assess the patient’s response to the methods used in patient-oriented therapy and evaluate individual potential of brain self-repair and stroke recovery. Future studies might include biomarkers as prognostic tools to estimate the brain ability to recover in a particular patient (neuroplasticity and angiogenesis activity) as well as a predictive tool for monitoring effectiveness of specific individually tailored rehabilitation interventions (Figure 1). In addition, the concept of biomarkers might have an impact on reducing a patient’s length of stay in hospital.

## 6. Conclusions

Recovery after a stroke-related brain injury is a very dynamic, complex, and multifactorial process, in which interaction among genetic, pathophysiologic, environmental, and therapeutic factors determines the overall recovery course. Therefore, the outcome is very often difficult to predict. Factors such as the extent of a stroke, spontaneous regeneration, angiogenesis, neuroplasticity, pharmacological treatment, and rehabilitation play important roles in a successful outcome, but significance of these factors should be assessed individually for every patient. The novel approach of personalized medicine and rehabilitation is intended to overcome those challenges in accordance with individually assessed factors that determine the achievement of the patient’s goals.

## Figures and Tables

**Table 1:** Features of selected, potential biomarkers of post-stroke regeneration. VEGF—vascular endothelial growth factor; Ang2—angiopoietin 2; BDNF—brain-derived neurotrophic factor; MMP9—matrix metalloproteinase 9.

Protein	Potential Advantages of Protein	References
VEGF	Risk factors do not affect the level of VEGF, indicating the possibility of widespread use among patients.	[26]
	The concentration of VEGF is significantly increased in post-stroke patients and remains augmented for 90 days, thus enabling the assessment of its changes during treatment.	[26,27]
	Rehabilitation may influence the level of VEGF, thus allowing the treatment progress to be monitored, and assess patient’s outcome.	[29]
Ang2	The *Ang2* expression in animal model may be linked with vasodilatation, destabilization of vessels, and detachment of pericytes in acute phase and the promotion of endothelial cell survival, and stabilization of vasculature in subacute phase of stroke, thus allowing the additional identification and differentiation of stroke phases among patients.	[34]
	Rehabilitation increases the Tie2 receptor, thus suggesting the role of Ang2-Tie2 in improving cerebral blood flow and microvasculature.	[35]
BDNF	Useful post-stroke recovery marker due to its broad neurotrophic activity and changes in its plasma concentration during recovery.	[46,47,54,55]
	The level of BDNF does not differ between sex, age and the initial severity of stroke.	[55]
	The correlation between low serum BDNF levels in the first day after stroke correlates with the poorer long-term functional prognosis.	[50,51,52]
	The positive correlation between serum BDNF level with function and mental status in post-stroke patients.	[53,56,57]
Irisin	Low concentration of irisin in hyper-acute stroke phase is associated with poor patients’ status (according to NIHSS scale) and outcomes three months after the ischemic episode, as well as increased mortality	[63,66,67]
MMP9	Increased level of MMP9 in hyper-acute phase of stroke is related to severity of stroke (according to NIHSS scale) regardless of other variables such as gender, age, and comorbidities (hypertension, diabetes, hyperlipidemia, etc.).	[70]
	High level of serum MMP9 in the acute phase of stroke is correlated with an increased risk of death or severe disability.	[72]
	High concentration of MMP9 in plasma correlates with increased risk of hemorrhagic transformation and infarct growth.	[74]

## Data Availability

The data presented in this study are available on request from the corresponding author.
